# Effect of restrictive cumulative fluid balance on 28-day survival in invasively ventilated patients with moderate to severe ARDS due to COVID-19

**DOI:** 10.1038/s41598-023-45483-8

**Published:** 2023-10-28

**Authors:** Ricardo Esper Treml, Tulio Caldonazo, Pedro Hilton A. Filho, Andréia L. Mori, André S. Carvalho, Juliana S. F. Serrano, Pedro A. T. Dall-Aglio, Peter Radermacher, João Silva Manoel

**Affiliations:** 1 Department of Anesthesiology and Intensive Care Medicine, Friedrich-Schiller-University, Jena, Germany; 2 Department of Cardiothoracic Surgery, Friedrich-Schiller-University, Jena, Germany; 3 Postgraduate Program, Department of Anesthesiology, University of São Paulo, Av. Dr. Arnaldo, 455, Cerqueira Cesar, São Paulo, SP 01246-903 Brazil; 4 Department of Anesthesiology, Servidor Público Estadual Hospital, São Paulo, Brazil; 5 Institute for Anesthesiological Pathophysiology and Process Development, Ulm University Hospital, Ulm, Germany

**Keywords:** Respiratory distress syndrome, Infection

## Abstract

This study aimed to evaluate the effect of two restrictive cumulative fluid balance (CFB) trends on survival and on major clinical outcomes in invasively ventilated patients with moderate to severe respiratory distress syndrome (ARDS) due to SARS-CoV-2. Prospective data collection was conducted on patients in the intensive care unit (ICU) originating from a tertiary university hospital. The primary outcomes were the risk association between the CFB trend during D_0_ to D_7_ and 28-day survival. The secondary outcomes were ICU mortality, in-hospital mortality, the need for invasive ventilation at D_28_, administration of vasoactive drugs at D_7_, time on invasive ventilation after D_7_, and length of ICU and hospital stay. 171 patients were enrolled in the study and divided according to their CFB trends during seven days of follow-up using model-based clustering [median CFB negative trend (n = 89) – 279 ml (− 664 to 203) and (n = 82) median CFB positive trend 1362 ml (619–2026)]. The group with CFB negative trend showed a higher chance of surviving 28-day in the ICU (HR: 0.62, 95% CI 0.41–0.94, p = 0.038). Moreover, this group had a reduced length of stay in the ICU, 11 (8–19) days versus 16.5 (9–29) days *p* = 0.004 and presented lower rates (OR = 0.22; 95% CI 0.09–0.52) of invasive ventilation after 28-days in the ICU. In patients invasively ventilated with moderate to severe ARDS due to COVID-19, the collective who showed a negative trend in the CFB after seven days of invasive ventilation had a higher chance of surviving 28 days in the ICU and lower length of stay in the ICU.

## Introduction

Patients with SARS-CoV-2 infection can express a broad spectrum of clinical manifestations, from asymptomatic to severe coronavirus disease 2019 (severe COVID-19) marked by a prominent acute respiratory distress syndrome (ARDS)^1^—the most frequent COVID-19-associated organ dysfunction^2^, pathophysiologically associated to the so-called COVID-19—hyperinflammation^3^.

Recent research links positive fluid balance in ICU and cumulative fluid balance (CFB) at ICU discharge with higher sepsis mortality^4,5^. In sepsis and ARDS patients, positive CFB worsens outcomes, including organ dysfunction and mortality, due to fluid-induced tissue edema^6,7^. Further, the Fluid and Catheter Treatment Trial (FACTT) study supports conservative fluid management, reducing ventilation duration and ICU stay without raising non-pulmonary organ dysfuntions^8^. Moreover, a negative fluid balance has been shown to predict patients' survival in septic shock^9^. In critical ill COVID-19 with ARDS, cumulative balance analysis on day 3 showed that negative balance was associated with higher chances of liberation from invasive ventilation^10^.

However, due to the lack of a clear definition of a restrictive fluid balance strategy, a broad spectrum of sub cohorts is present inside this collective^8,9,11^.Thus, which sub cohort inside a restrictive fluid strategy has a better outcome-related performance in the ICU, in special, in ARDS patients due to COVID-19 remains not completely elucidated. Therefore, our hypothesis is that patients with a tendency to negative CFB 7 days after the start of invasive ventilation will have a better chance of survival in the ICU. To test this hypothesis, we performed a survival analysis prospectively using a model-based clustering to evaluate the effect of restrictive trends in CFB seven days after initiation of invasive ventilation on 28-day survival in the ICU and other key outcomes.

## Materials and methods

### Study design

We conducted a prospective observational single-center cohort study in the ICU of a tertiary hospital. This study was performed from May 2020 to December 2020, before the start of vaccination against SARS-CoV-2.

### Ethical approval and consent to participate

The hospital's ethical committee approved the study protocol (ethical approval number: 4.022.319 CEP, IAMSPE Ethical Committee, approval date: May 2020). It was conducted according to the STROBE-Guidelines for prospective observational studies^12^ and respecting the Helsinki Declaration. Informed consent was obtained from the patient’s relatives or legal representatives for all included patients. From survivors, informed consent was obtained after release from the ICU. The clinical chemistry and laboratory diagnostic of the involved hospital provided analysis of blood samples. For all study time points, the worst laboratory and clinical values within the preceding 24-h interval were considered for the final analysis.

### Access of organ dysfunction and estimation of ICU mortality

Organ dysfunction was assessed at all study time points using the Sequential Organ Failure Assessment (SOFA) score^13^. To estimate ICU mortality, we used the simplified Acute Physiology Score (SAPS 3) on admission to the ICU.

### Study population and eligibility

We screened and selected patients older than 18 years old with confirmed infection due to SARS-CoV-2 with a positive polymerase chain reaction (PCR) and a confirmed diagnosis of COVID-19 disease and moderate to severe ARDS according to the Berlin definition^14^, which required endotracheal intubation and mechanical ventilation within 24 h of admission at the intensive care unit (Patents with COVID-19 and moderate-severe ARDS requiring mechanical ventilation). None of the patients included in this study was vaccinated against SARS-CoV-2. All patients received the standard of care according to the Surviving Sepsis Campaign Guidelines on the Management of Critical ill Adults with COVID-19^15^ and were ventilated according to the guidelines for protective mechanical ventilation in patients with ARDS using low tidal volume (*V*_T_: 4–6 ml/kg PBW), targeting driving pressures lower than 15 cmH_2_O with individualized positive end-expiratory pressure (PEEP)^16^. There was no institutional protocol for a fluid restrictive strategy, yet in general there was a clinical practice of restricting fluids during the care of these patients. The detailed exclusion criteria are exposed in the Supplemental Digital Content S1.

### Data collection and follow-up

All patients were followed until hospital discharge or death. The data collection was performed prospectively from the electronic patient healthy record (REDCap^®^). The demographic data were obtained after the enrolment in the study. Clinical and laboratory assessments were performed at all 3 study time points (D_0_, D_3,_ and D_7_). D_0_ was considered as baseline data after intubation and the start of invasive ventilation. D_3_ was defined as three days after initiation of invasive ventilation, and D_7_ as seven days after invasive ventilation. Acute kidney injury (AKI) was defined according to the KDIGO-Guidelines (not graded) considering the baseline creatinine from D_0_ as any of the following increases in serum creatinine ≥ 0.3 mg/dl within 48 h or an increase in serum creatinine ≥ 1.5 times baseline or < 0.5 ml/kg/h for 6 h during D_0_–D_7_ (not graded)^17^. The clinical assessment of pulmonary function, patients' hemodynamics, and laboratory parameters are summarized in the Supplemental Digital Content S1.

### Model-based clustering groups allocation

To minimize a dichotomization bias including some patient subgroups in the impropriated cohort it was chosen the K-mean using the trend mean as longitudinal vector trajectory to perform the correct allocation of subjects in the cohorts reducing the confounding bias of heterogeneous trends of CFB within a group of patients. To mitigate the risk of improper patient allocation into unsuitable clusters and to gauge the cohesiveness of the group, we leveraged the Silhouette score. This score yielded a value of 0.93, signifying a substantial degree of segregation among the groups, thus indicating a robust clustering arrangement (Supplemental Digital Content Fig. S1). Therefore, we use a model-based clustering group allocation based on their CFB predominant trend from D_0_ to D_7_ (> 50% of the 7 days)^18^ using k-means clustering in 100 sub-samples (n = 2/3 of the corresponding original sample size) within the sample of patients with complete data on the fluid balance for days 1 to 7. We restricted the number of possible clusters (*k*) to 2–15, and the optimum was obtained using the Calinski-Harabasz index (cluster boot function, *R FPC package*^19,20^. The K-mean cluster approach was based on the daily CFB within 7 days after initiation of invasive ventilation using the predominant trend from D_0_ to D_7_ as longitudinal vector trajectory for the clustering. Thus, after the model-based clustering, the collective was divided into two groups according to their CFB trend^18–20^ (Supplemental Digital Content Table S3 and Fig. S2).

### Outcomes

The primary outcome is the risk association between the CFB trend during D_0_ to D_7_ and 28-day survival. As secondary outcomes, we evaluated the association of CFB trend during D_0_ to D_7_ and the need for invasive ventilation at day 28, administration of vasoactive drugs at D_7_, time on invasive ventilation after D_7_, ventilation and vasopressor free days, length of ICU stay, in-hospital mortality, ICU mortality and hospital stay (ICU-LOS and Hospital-LOS). Explorative we addressed the incidence of AKI at D_7._

### Statistical analyses

For the statistical analyses of the continuous demographic, clinical, and laboratory data, values were summarized as means (± SD) and median (Q1/3). We reported their absolute and relative absolute and relative frequencies for categorical variables. The distribution of the variables was tested using the Shapiro–Wilk test. To compare the baseline demographic D_0_ data between the groups we applied Mann–Whitney *U* tests for the continuous variables and χ^2^-tests for categorical variables. For the direct comparison between D_0_ and D_7_ from each group separately, we used Wilcoxon signed rank tests for the comparison between continuous variables and χ^2^-tests for the comparison between categorical variables. For the comparison of clinical and laboratory data between groups at D_0_, D_3_, and D_7_, we used generalized estimating equations (GEE) models with marginal Poisson distribution and an identity link function, assuming a first-order autoregressive (AR1)^21^ correlations between assessment times. The results were followed by Bonferroni multiple comparisons to identify the differences between groups and time points when significant^22,23^.

In the analyses of our primary outcomes, we conducted a survival analysis using the CFB trend as a dependent variable for outcomes estimating the cumulative-event probabilities. We calculated adjusted Hazard ratio (HR) and 95% confidence in the Kaplan–Meier survival by Cox Model stepwise, and the odds ratio were calculated using a multiple logistic regression stepwise model adjusted for possible confounders (age, gender, BMI, Charlson-comorbidity index, and SAPS 3 baseline [D_0_]). Variables with multicollinearity were removed from the final analysis. For the risk analysis of our secondary outcomes, we calculated the odds ratio and 95% confidence using a multiple logistic regression adjusted for possible confounders using the CFB trend as a dependent variable, excluding variables with multicollinearity from the final analysis. The logistic regression models were tested using a generalized Hosmer–Lemeshow goodness-of-fit test^24^.

Statistical analysis was performed with IBM SPSS 26 (IBM Corporation, Armonk, NY, USA) and Graphpad Prism 7.05 (Graphpad Software Inc., San Diego). We applied a significance level of 5% and reported two-sided p-values.

## Results

**Figure 1:**
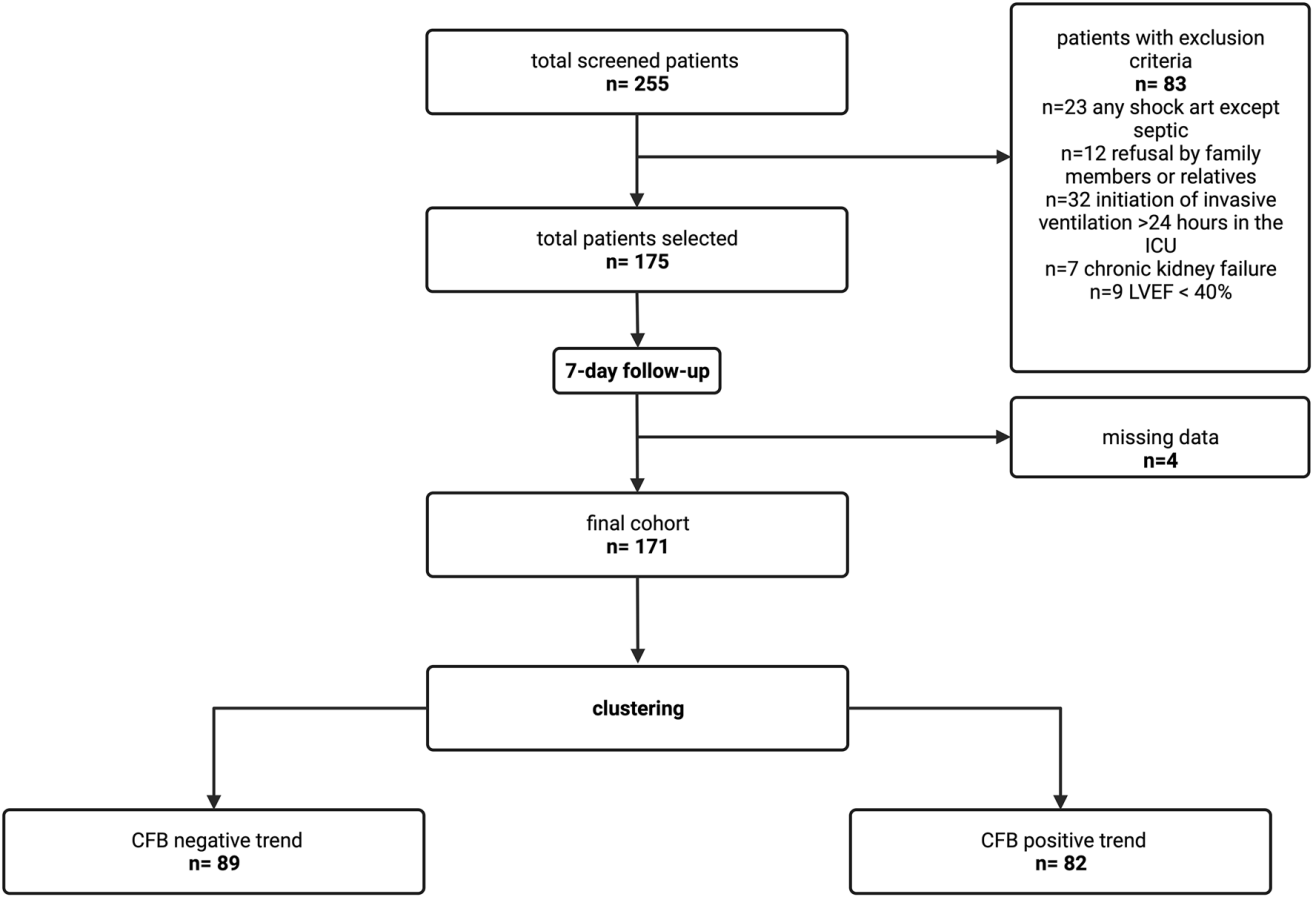
Study design and overview of the patient inclusion and analytical cohorts. *CFB* cumulative fluid balance, *ICU* intensive care unit, *LVEF* left ventricle ejection fraction.

Figure 1 describes the study design. Initially, 258 patients were screened. After removing patients based on exclusion criteria and patients who did not present complete data before D_7_, 171 patients were included in the final cohort. Using a model-based clustering group allocation based on their CFB trend from D_0_ to D_7_, 89 patients were clustered into the CFB negative trend median -279 ml group and 82 patients into the CFB positive trend group median 1362 ml (Supplemental Digital Content Fig. S3).

### Baseline characteristics

Table 1 shows the descriptive demographic data. Both studied groups had similar demographic characteristics, including age, sex, BMI, Charlson-comorbidity index, SOFA and SAPS III scores, ventilatory and laboratory parameters, and need for vasopressor drugs. In summary, both patient cohorts were median-aged (66 years), overweight (BMI ≥ 26 kg/m2), mostly male, and had comparable preexisting comorbidities. Clinically, they exhibited a similar degree of organ dysfunction (SOFA score of 6) and a similar level of pulmonary dysfunction, with a median oxygenation index of 110 in the CFB negative trend group and 100 in the CFB positive trend group, both meeting the criteria for moderate ARDS according to the Berlin criteria^14^.

**Table 1:** Descriptive demographic data of the group values*.*

Variables	CFB negative trend (−) D_0_	CFB positive trend (+) D_0_	*p*-value
	(n = 89)	(n = 82)	
Age [years]; mean ± SD	64.6 ± 12.1	66.3 ± 10.0	0.323
Median (Q1–Q3)	66 (59–72)	67.5 (60–74)	
Male sex; n [%]	56 (62.9%)	44 (53.6%)	0.219
Weight [kg]; mean ± SD	85.0 ± 19.1	81.1 ± 16.6	0.249
Median (Q1-Q3)	80.0 (71–96)	78 (70–90)	
Height [m]; mean ± SD	1.68 ± 0.09	1.68 ± 0.10	0.915
Median (Q1-Q3)	1.68 (1.61–1.76)	1.69 (1.61–1.77)	
BMI [kg/m^2^]; mean ± SD	29.8 ± 5.8	28.4 ± 4.7	0.175
Median (Q1-Q3)	28.67 (26–32.4)	27.7 (25.3–31)	
Charlson–comorbidity index; mean ± SD	1.4 ± 0.5	1.5 ± 0.9	0.190
Median (Q1–Q3)	1 (0–3)	1 (0–3)	
Baseline admission parameters			
SOFA Score; mean ± SD	6.1 ± 2.3	5.9 ± 2.4	0.445
Median (Q1–Q3)	6 (4–7)	5 (4–7)	
SAPS III Score; mean ± SD	55.4 ± 12.3	57 ± 11.8	0.465
Median (Q1–Q3)	55 (47–64)	58 (47.5–64.5)	
Oxygenation index; mean ± SD	124.5 ± 66	124.2 ± 72	0.960
Median (Q1–Q3)	110 (79–146)	100 (80–143)	
PaO_2_ [mmHg]; mean ± SD	130 ± 68.3	131 ± 73.0	0.821
Median (Q1–Q3)	118.1 (78–147)	118.7(80–155)	
FiO_2_ [%]; mean ± SD	89.4 ± 18.3	90 ± 16.7	0.850
Median (Q1–Q3)	100 (80–100)	100 (80–100)	
Creatinine [mg/dL]; mean ± SD	1.12 ± 0.71	1.4 ± 1.4	0.101
Median (Q1–Q3)	1 (0.75–1.25)	1 (0.8–1.6)	
Mean arterial pressure; mean ± SD	83.5 ± 15	83.4 ± 18.5	0.837
Median (Q1–Q3)	81 (74–95)	80 (74–93.7)	
Lactate [mmol/L]; mean ± SD	1.94 ± 0.64	2.12 ± 0.90	0.390
Median (Q1–Q3)	1.74 (1.52–2.3)	1.90 (1.56–2.43)	
Hemoglobin [g/dL]; mean ± SD	12.3 ± 1.87	12.2 ± 2.0	0.796
Median (Q1–Q3)	12.5 (11.4–13.5)	12 (11–13.7)	
Lung injury^#^ < 50%; n (%)	54 (60.6%)	44 (53.6%)	0.439^#^
Lung injury^#^ ≥ 50%; n (%)	35 (39.3%)	38 (46.3%)	
Administration of vasopressor drugs* n (%)	28 (31%)	29 (35%)	0.628*

Values summarized as mean ± SD and median Q1/3 (first and third quartile). p-value was calculated by the Mann–Whitney-U test for continuous variables and the ^#^Chi-square test for categorical variables, respectively (p < 0.05).

*BMI* body-mass-index, *FiO*_*2*_ fraction of inspired oxygen, *SAPS III* simplified acute physiology score, *SOFA Score* sequential organ failure assessment score, *PaO*_*2*_ partial pressure of oxygen in the arterial blood.

^#^Based on chest computed tomography.

*Any use of dopamine, vasopressin, epinephrine, or norepinephrine for more than 1 h.

### Clinical and laboratory parameters

**Figure 2:**
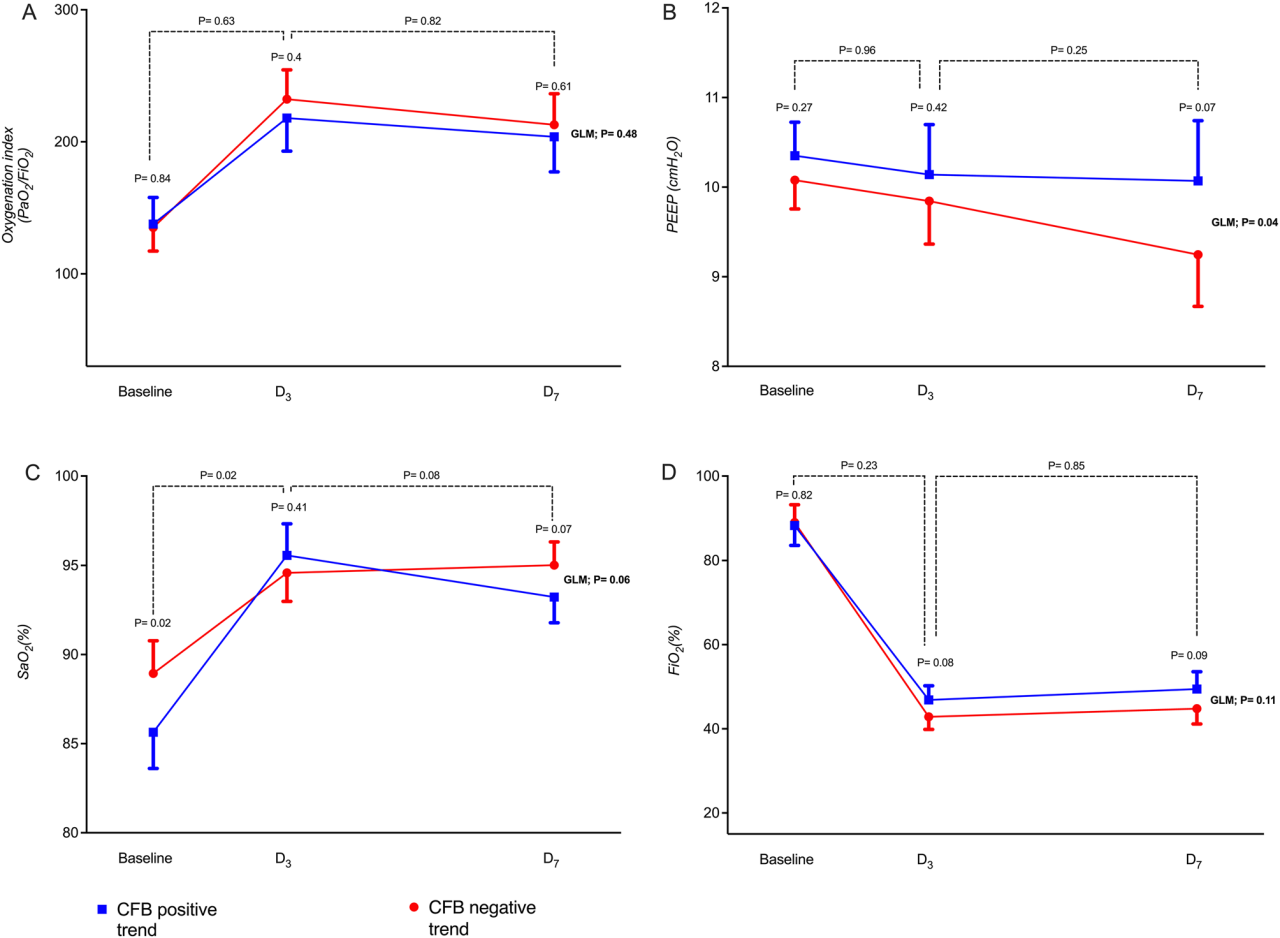
Comparison of ventilatory and laboratory parameters in different study time points (D_0_, D_3,_ and D_7_) between the cohorts: (**A**) oxygenation index, (**B**) arterial oxygen saturation (SaO_2_), (**C**) positive end-expiratory pressure (PEEP) and (**D**) fraction of inspired oxygen (FiO_2_).

Table 2 demonstrates the clinical and laboratory parameters of the studied groups at study times D_0_ and D_7_. In the CFB negative trend group, comparing their baseline to D_7,_ the analyzed values presented not a statistically significant difference regarding the SOFA score but significantly increased creatinine and lactate plasma levels and a significant decrease in MAP and serum hemoglobin. Similarly, in the CFB positive trend group, the comparison of the D_0_ and D_7_ values also presented no statistically significant difference in the SOFA Score. However, in this group, there was a significant increase in serum creatinine and a decrease in serum hemoglobin. Comparing the D_7_ data of each group, there was no statistically significant difference in the SOFA Score, creatinine, and lactate levels. Still, the CFB negative trend group presented significantly lower MAP and higher hemoglobin values than the CFB positive trend group. An exploratory comparison was made at D_3_ between laboratory, clinical and CFB trend of both cohorts (Supplementary Table S5, Fig. 2, D_3_ time point). In summary, at D_3_ the CFB negative trend presented higher levels of MAP than the positive trend, However, interestingly, the group with a positive tendency had higher creatinine levels.

**Table 2:** Comparison of clinical and laboratory data of the collectives at baseline *vs.* D_7_ and between groups at D_7_.

Variables	CFB negative trend (−)	CFB negative trend (−)	*p*	CFB positive trend (+)	CFB positive trend (+)	*p*
	D_0_ (n = 89)	D_7_ (n = 89)		D_0_ (n = 82)	D_7_ (n = 82)	
SOFA score, mean** ± **SD	6.1 ± 2.3	6.4 ± 2.4	0.919	5.9 ± 2.4	6.2 ± 2.7	0.681
Median (Q1–Q3)	6 (4–7)	6 (4–8)		5 (4–7)	6 (4–8)	
Creatinine [mg/dL], mean** ± **SD	1.12 ± 0.71	1.80 ± 1.20	** < 0.001**	1.4 ± 1.4	2.1 ± 1.6	** < 0.001**
Median (Q1–Q3)	1 (0.75–1.25)	1.6 (0.9–2.7)		1 (0.8–1.6)	1.5 (0.9–3)	
Mean arterial pressure, mean** ± **SD	83.5 ± 15	77.1 ± 15.1	**0.026**	81.6 ± 18.1	80.7 ± 12.5	0.257
Median (Q1–Q3)	81 (74–95)	77 (67–86)		80 (74–92.7)	79.5 (72–87)	
Lactate [mmol/L], mean** ± **SD	1.94 ± 0.64	2.77 ± 3.8	** < 0.001**	2.12 ± 0.93	2.46 ± 1.1	0.681
Median (Q1–Q3)	1.74 (1.52–2.3)	2.15 (1.7–2.9)		1.90 (1.56–2.43)	2.2 (1.9–2.5)	
Hemoglobin [g/dL], mean** ± **SD	12.3 ± 1.87	11.16 ± 1.70	** < 0.001**	12.2 ± 2.0	10.17 ± 1.80	** < 0.001**
Median (Q1–Q3)	12.5 (11.4–13.5)	11 (10.1–12.4)		12 (11–13.7)	10.1 (8.6–11.8)	
Comparison of D_7_ vs. D_7_						
SOFA score, mean** ± **SD	6.4 ± 2.4			6.2 ± 2.7		0.436
Median (Q1–Q3)	6 (4–8)			6 (4–8)		
Creatinine [mg/dL], mean** ± **SD	1.80 ± 1.20			1.5 (0.9–3)		0.240
Median (Q1–Q3)	1.6 (0.9–2.7)			1.5 (0.9–3		
Mean arterial pressure, mean** ± **SD	76.6 ± 15.0			81.5 ± 12.03		**0.043**
Median (Q1–Q3)	77 (66.5–86)			80 (74–87)		
Lactate [mmol/L], mean** ± **SD	2.77 ± 3.8			2.46 ± 1.1		0.620
Median (Q1–Q3)	2.15 (1.7–2.9)			2.2 (1.9–2.5)		
Hemoglobin [g/dL], mean** ± **SD	11.24 ± 1.65			10.12 ± 1.80		** < 0.001**
Median (Q1–Q3)	11 (10.1–12.4)			10.1 (8.6–11.8)		
Cumulative fluid balance trend [ml], mean** ± **SD	− 298 ± 612			1405 ± 930		** < 0.001**
Median (Q1–Q3)	− 279 (− 664 to 203)			1362 (619–3773)		

Values summarized as mean ± SD and median Q1/3 (first and third quartile).p-value was calculated by Wilcoxon signed rank test for the comparison between baseline and D_7_ continuous variables of each group. The comparison von D_3_ vs. D_3_ was mad with Mann–Whitney test.

*SOFA* score: sequential organ failure assessment score, *PaO*_*2*_ partial pressure of oxygen in the arterial blood, *PEEP* positive end-expiratory pressure, *p* p-value (< 0.05 marked in bold).

Figure 2 shows the ventilatory and laboratory parameters at different study time points. Both groups showed a dynamic improvement in respiratory parameters such as reduced FiO_2_, increased oxygenation index, and arterial oxygen saturation. However, the group CFB with a negative trend presented lower PEEP levels on D_7_ (Fig. 2B). The two groups showed similar oxygenation index and FiO_2_ values with no statistical difference in all study time points (Fig. 2A,D).

### Primary and secondary endpoints

**Figure 3:**
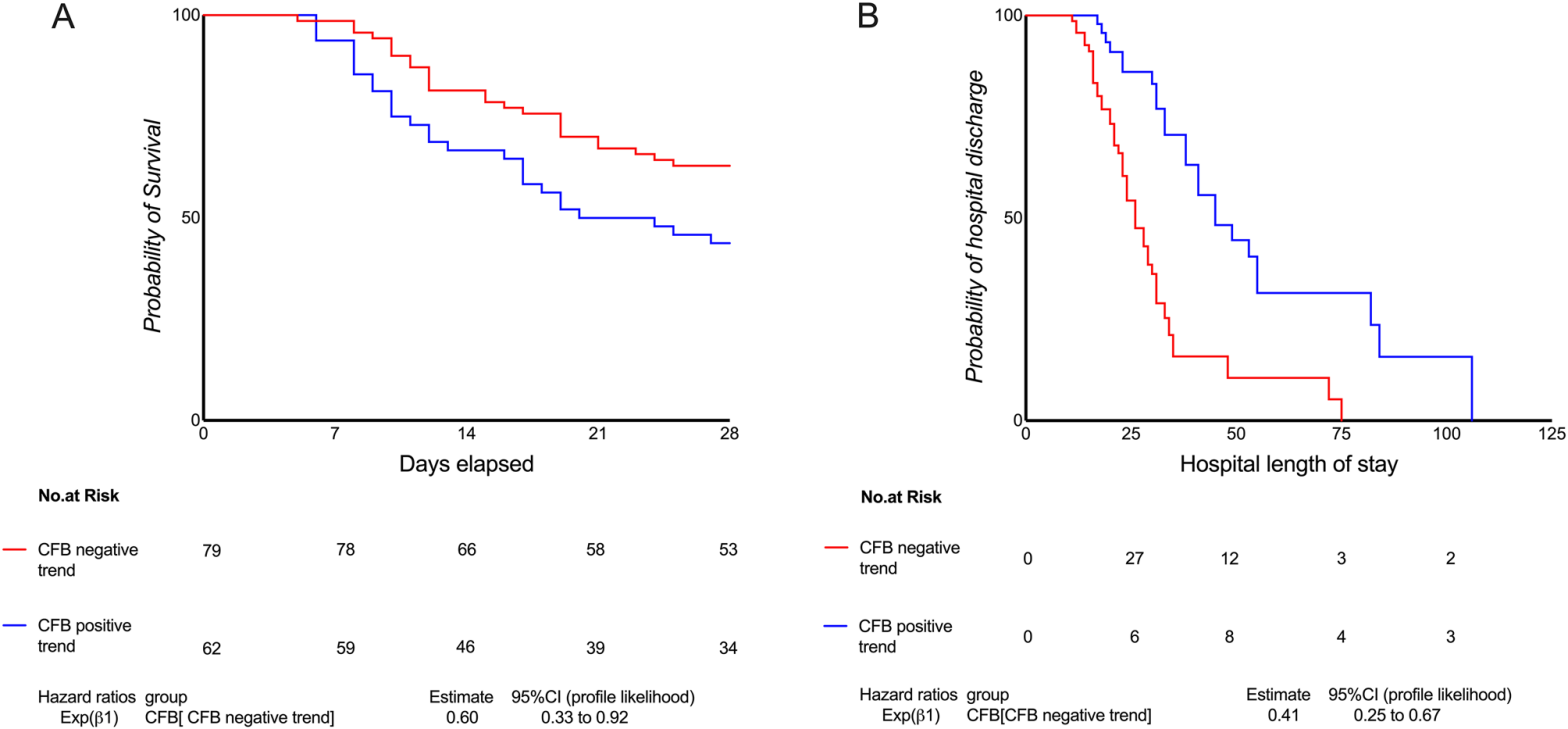
Kaplan–Meier with the estimated cumulative probability of 28-day survival. Cumulative Fluid Balance (CFB) at D_7_, 95% Confidence Interval (95%CI). Central Picture: Graphical abstract summarizing the main findings.

Eighty-nine patients in the CFB negative trend group and 82 in the CFB positive trend group were in the follow-up. 28-day mortality was 46% *vs.* 64% and the ICU mortality 30% vs. 46% respectively (Fischer’s exact test *p* = 0.021 and p = 0.003). However, the in-hospital mortality had no significant difference 40.4% vs. 55% (Fischer’s exact test *p* = 0.059). Figure 3 shows the Kaplan–Meier survival curve for the 28-day follow-up in the ICU. The unadjusted survival hazard in the CFB negative trend group was HR: 0.62, 95% CI 0.41–0.94, *p* = 0.032. After adjustments to minimize the effect of confounding variables, the CFB negative trend group maintained a higher chance of survival 28-day in the ICU (adjusted Odds Ratio: 0.70, 95% CI 0.24–0.98, *p* = 0.038). Table 3 summarize the secondary outcomes. The CFB negative trend group showed decreased risk of requiring invasive ventilation at D_28,_ and higher odds to need vasopressors drugs at D_7_ and being more days on use of vasopressor in comparison with the CFB positive trend group. This group showed also decreased ICU LOS but no significant difference in the Hospital LOS. The incidence of AKI at D_7_ didn’t differ between the groups (50.56% CFB negative trend and 40.2% CFB positive trend Fischer’s exact test *p* = 0.219).

**Table 3:** Secondary outcomes summary.

Outcomes	CFB negative trend (−)	CFB positive trend (+)	Adjusted odds ratio		
	(n = 89)	(n = 82)	p*	Odds ratio	95% CI
Need of invasive ventilation at D_28_*, n (%)	9 (10%)	13 (15%)	** < 0.001**	0.22	0.09–0.52
Administration of vasoactive drugs at D_7_*, n (%)	52 (66%)	14 (22.6%)	** < 0.001**	3.0	1.90–4.93
Ventilation free days					
Mean ± SD	16 ± 7	17 ± 7.7	0.655		
Median (Q1–Q3)	17 (12.2–20)	17.5 (11–21.2)			
Vasopressor free days					
Mean ± SD	21.4 ± 3.7	22.25 ± 3.8	**0.044**		
Median (Q1–Q3)	21 (19–25)	24 (21–25)			
Time on invasive ventilation after D_7_^#^					
Mean ± SD	15.17 ± 9.3	20.4 ± 16.3	**0.048**^**#**^		
Median (Q1–Q3)	12 (9–19)	14.5 (9–25.5)			
In-hospital mortality n (%)	36 (40.4%)	45 (55%)	0.059°		
ICU mortality	27 (30%)	38 (46%)	**0.003**°		
ICU LOS^#^					
Mean ± SD	14.6 ± 10.2	21.6 ± 17.4	**0.004**^**#**^		
Median (Q1–Q3)	11 (8–19)	16.5 (9–29)			
Hospital LOS^#^					
Mean ± SD	23.8 ± 13.7	32 ± 23.5	0.082		
Median (Q1–Q3)	21 (15–30)	25 (13–45)			

The total number of patients is summarized as n, number (percentage). Vasopressor: use for more than 1 h of Dopamine > 5 µg/kg/min or epinephrine ≤ 0.1 µg/kg/min or norepinephrine ≤ 0.1 µg/kg/min or dopamine > 15 µg/kg/min or epinephrine > 0.1 µg/kg/min or norepinephrine > 0.1 µg/kg/min. Ventilation: any duration of invasive ventilation ^#^p-value was calculated by the Mann–Whitney U test for continuous, p° Fischer’s exact test ex p* value for the secondary outcomes and was estimated using multiple logistic regression adjusted for age, gender, BMI, Charlson-comorbidity index, and SAPS III baseline.

*95% CI* confidence interval, *p* p-value (< 0.05 marked in bold), *D*_*7*_ 7 days after initiation of invasive ventilation, *LOS* length of stay, *ICU* intensive care unit.

*The logistic regression models were tested using a generalized Hosmer–Lemeshow goodness-of-fit test (> 0.05).

## Discussion

In this prospective single-center observational study in patients with moderate to severe ARDS due to SARS-CoV-2, one could observe a higher hazard of 28-day survival in patients with CFB with a negative trend seven days after invasive ventilation. It is noteworthy that our patient cohort demonstrated similar demographic characteristics to those previously described for individuals afflicted with severe COVID-19. In our study, both collectives were predominantly male, over 60 years old, and overweight. Older age, male gender, and being overweight are risk factors for developing severe and critical COVID-19 disease and sepsis from other etiology^25–29^.

In critically ill patients and in patients undergoing major surgeries fluid overload is associated with worse outcomes increasing their morbidity and mortality^11,30^. Data obtained from observational studies that included septic patients and patients with critical neurological diseases indicate a deterioration in clinical outcomes in presence of fluid overload^6,31^. This deterioration is manifested by prolonged stays in the ICU, increased in-hospital mortality rates, and a progression of organ dysfunctions, including the worsening of lung function^6,30,32^. In a different clinical context, involving patients with moderate to severe ARDS due to the SARS-CoV-2 virus, we were able to show a worsening in the clinical outcome of patients who tended to have a positive fluid screen within 7 days. In the other hand, patients with a negative CFB trend had better outcomes. This was translated to an increased likelihood of survival and a reduced duration of stay in the ICU. Interestingly, this group had less time on mechanical ventilation after day 7 and need of invasive Ventilation after 28 days, although there was no difference in the days free of mechanical ventilation and in the in-hospital mortality.

Currently, there is no established consensus for guiding fluid management in patients with ARDS^33–35^. There is a logical propensity to prioritize fluid restriction since the pathophysiological mechanism to explain the improvement of hematosis by restricting the fluid input is the reduction of tissue, cellular, interstitial, and alveolar edema caused by the lesion of the alveolar-capillary bed by the inflammatory process^36,37^. However, the absence of a clearly defined restrictive fluid management strategy in sepsis^4,5,9,38^ in patients with ARDS^33,35,39^, and now in patients with ARDS caused by SARS-CoV-2^40^, makes it challenging to compare various studies and their findings. This challenge arises from the lack of consensus regarding the specific limits for defining a fluid balance as restrictive and the absence of a universally accepted clinical protocol^34,41^.

Nonetheless, moderate to severe ARDS induced by SARS-CoV-2 closely resembles non-COVID-19 ARDS physiologically, making findings from COVID-19 ARDS studies relevant to ARDS from various causes^42^.Improved lung function and ventilation parameters have been demonstrated in studies evaluating restrictive fluid replacement therapy in ARDS due to sepsis^43,44^. We could observe these findings in invasively ventilated patients with moderate to severe ARDS due to COVID-19-sepsis. Both the CFB negative and positive trends showed improvement in oxygenation and ventilation parameters when comparing the evolution of their baseline, D_3,_ and D_7_ values.

For instance, two large trials shared similar findings. Firstly, the FACTT trial showed the benefit of the conservative fluid strategy over a liberal approach. That trial showed significant oxygenation and ventilatory improvements without an increase in other organ dysfunction but no significant reduction in 60-day mortality was found. Some points may explain the mortality difference found between the two restrictive trends of CFB in our study: (1) We studied a group of patients with ARDS due to COVID-19-sepsis, unlike the FACTT trial, where only 14.3% of the patients had pneumonia or sepsis; (2) the two groups evaluated by our study are restrictive, making our data only comparable to the restrictive arm of the FACTT trial; (3) the baseline characteristics such as the predominance of elderly and obese patients of patients with ARDS due to COVID-19 differed from that found for ARDS of other origins, as the studied collective in the FACTT trial showed (4), FACTT trial had a interventional nature with an established protocol for fluid restriction differs from our present observational study. and finally (5) We evaluate mortality within a brief timeframe of 28 days.

An insight from the PRoVent-COVID-19 study evaluated the association between early CFB and successful liberation from invasive ventilation in COVID-19 ARDS patients. In terms of the studied collective, the baseline characteristics of our collective of patients with ARDS due to COVID-19-sepsis resemble demographically, clinically and laboratory the collective presented by the sub-analysis from the PRoVent-COVID-19 study. This study analyzed three groups of patients: higher, intermediate, and lower groups, having a median CFB of 1.98 L (1.27–7.72 L), 0.78 L (0.26–1.27 L), and − 0.35 L (− 6.52 to 0.26 L), respectively. In summary, this study found a risk of a lower likelihood of successful release from invasive ventilation on day 28 in the group with higher CFB on day 3. In addition, this group showed longer invasive ventilation time and hospital length of stay. Still, no difference was found regarding the incidence of AKI, 28-day mortality, and length of stay in the ICU^40^.

In the present study, comparing the two CFB trends, we were able to show a higher hazard of 28-day survival and lower length of stay in the ICU. Although our study observed less need for invasive ventilation in the restrictive CFB, an essential difference in study design may explain the difference in survival and organ dysfunction in our study like the assessment of the risk association between the CFB trend and 28-day mortality over a more extended observation period (seven days). A fact that can more comprehensively characterize the clinical progression of patients of this category pertains to the noteworthy average duration of mechanical ventilation, which spans 13.5 days^10,45^. Employing an observation window of 7 days could offer a more accurate portrayal of the patient's trajectory and enable the discernment of the impacts of fluid-restrictive strategies. It is worth noting that a substantial proportion of extant research studies typically evaluate the effects of these strategies over a mere 3-day interval^10^. This relatively short observational period may not adequately elucidate the potential advantages associated with a restrictive strategy, since both groups are still in the post-acute phase due to possible fluid overload, characteristic of the acute phase of critically ill patients^30,32,46^. Our own analysis corroborates this contention, as we observed that, within the initial 3 days of monitoring, there were no discernible or statistically significant disparities in the clinical parameters pertinent to respiratory outcomes between the two cohort.

Considering our observations regarding 28-day survival and mortality, given the absence of a distinction in in-hospital mortality within our study, it becomes imperative that future randomized investigations and meta-analyses are conducted to assess the potential advantages of fluid-restrictive interventions and evaluation of CFB trends in patients with ARDS, particularly in cases of viral sepsis, such as those resulting from SARS-CoV-2 infection.

Restrictive fluid strategies have being associated in critical ill patients with higher need of vasopressors and development of AKI^47^. Indeed, our study found higher need of vasopressor in patients with CFB with negative trend. Restrictive fluid management in critically ill patients with ARDS has been associated with a decline in intravascular pressures and increased use of vasopressors to maintain adequate hemodynamics^40^. On the other hand, the group with a negative trend showed higher lactate levels, which may indicate a worsening in tissue perfusion. A fact that may be associated with greater organ dysfunction^48^. Nonetheless, regarding the incidence of AKI, no statistical difference was found using the ungraded KDIGO criteria for AKI^17^ (50.5% in the CFB negative trend group and 40.2% in the CFB positive group), equally finding to PRoVent-COVID-19 study^40^. Our findings align with a large observational study showing similar incidence of AKI in hospitalized patients with COVID-19.

### Limitations

Our findings should be taken into consideration in regard of the study limitations. First, we conducted a single-center study, making the projection of our data to other centers difficult. In addition, it was an observational, not randomized, study without interventions. Moreover, the lack of an institutional protocol for fluid restriction restricts the application of results in other centers. Further, the effect of different diuretic drugs including the different off-label therapies for COVID-19 on outcomes was not assessed. An important fact to mention is that we did not grade acute renal dysfunction, making the evaluation of the effect or risk associations of this variable difficult. Moreover, we did not evaluate the parameters of fluid responsiveness and intracardiac pressures by echocardiography. Finally, the causal etiology of need for vasopressor was not addressed as well as the fact that the inclusion of some patients with a slight reduction in LVEF (between 40 and 50%) could be a potential confounding factor. However, taking in consideration our findings, this study can serve as a basis for multicenter clinical randomized studies based on the CFB trend in the assessment of the effect of restrictive fluid balance in patients with moderate to severe ARDS.

## Conclusion

In patients invasively ventilated with moderate-severe ARDS due to COVID-19, the collective who showed a negative trend inside the CFB after seven days of invasive ventilation had a higher chance of surviving 28 days and lower length of stay in the ICU. Further, studies are needed to confirm these benefits, especially in patients with ARDS of viral origin.

### Supplementary Information

Supplementary Information.

## Data Availability

All patient-related work data or statistical analysis is available for the next 10 years for free consultation. The datasets used and/or analysed during the current study available from the corresponding author on reasonable request.
