# Supplementary material for: Effect of restrictive cumulative fluid balance on 28-day survival in invasively ventilated patients with moderate to severe ARDS due to COVID-19
Source: Sci Rep. 2023 Oct 28;13:18504. doi: 10.1038/s41598-023-45483-8 (PMC10613222; doi:10.1038/s41598-023-45483-8)
Supplement: Supplementary file 1 — Supplementary Information. [file 41598_2023_45483_MOESM1_ESM.docx]

**Supplemental Digital Content**

**Supplementary Table 1**. Detailed exclusion criteria.

**Supplementary Table 2.** Clinical assessment of the pulmonary function and hemodynamics.

**Supplementary Table 3.** Detailed model-based clustering group allocation strategy.

**Supplementary Table 4.** Patient study power analysis.

**Supplementary Table 5.** Comparison of clinical and laboratory data of the collectives at baseline *vs.* D_3_ and between groups at D_3_.

**Supplementary Figure 1.** Cumulative fluid balance trend based on the divided groups after the model-based clustering visual distribution of clusters using dispersion.

**Supplementary Figure 2.** Example of vector trajectory for clustering using k-mean Trend

**Supplementary Figure 3.** Cumulative fluid balance trend based on the divided groups after the model-based clustering.

**Supplementary Figure 4.** Kaplan-Meier with the estimated cumulative probability of 28-day survival using CFB trend at D_3_ as independent variable.

**Supplementary References**

**Supplementary Table 1**. Detailed exclusion criteria.

| **Patients were not selected for this study if any of the following criteria were present:** |
| --- |
| Patients <18 years |
| Refusal by family members or relatives not reached within 24 hours of initiation of mechanical ventilation |
| Pregnancy or breastfeeding patient |
| Initiation of mechanical ventilation >24 hours after ICU admission |
| Patients with terminal chronic kidney injury according Kidney Disease Improving Global Outcomes Guidelines (KDIGO-Guidelines) defined as abnormalities of kidney structure or function |
| Present >3 months with a category of glomerular filtration ratio (GFR) ≤59 ml/min/1.73m2 |
| Patients with heart failure with left ventricular ejection fraction less than 40% on the echocardiography |
| Patients with chronic liver disease CHILD B or C |
| Patients who developed circulatory shock during the follow-up |

**Supplementary Table 2.** Clinical assessment of the pulmonary function and hemodynamics.

| **Pulmonary function** |
| --- |
| The clinical assessment of the pulmonary function was performed by analyzing the oxygenation index (ratio between the partial pressure of oxygen [PaO_2_] and the fraction of inspired oxygen [FiO_2_]) according to the severity classification of ARDS of the Berlin definition (1). Therefore, the patient's FiO_2_ (%) was documented, and the PaO_2_ arterial was collected at the time of the FiO_2_ documentation. For the analyses of the laboratory data (PaO_2_, **creatinine, lactate, hemoglobin),** blood samples were collected at all three study time points **and were analyzed on the same day. Acute kidney injury (**AKI) was defined according to the KDIGO-Guidelines (not graded) considering the baseline creatinine from D_0_ as any of the following increases in serum creatinine ≥0.3 mg/dl within 48 hours or an increase in serum creatinine ≥1.5 times baseline or <0.5 ml/kg/h for 6 hours during D_0_ to D_7_ (not graded) (2). |
| **Hemodynamics** |
| Clinically, patients' hemodynamics were evaluated through their mean arterial pressure (MAP) and the administration of any vasopressor (dopamine, vasopressin, epinephrine, or norepinephrine), regardless of its dose. The vasopressor administration was considered in the final analysis and documented as present when used for more than 1 hour to maintain a MAP of ≥65 mmHg. |

**Supplementary Table 3. Cumulative fluid balance calculation.**

| The CFB calculation was performed daily. For the fluid balance calculation, we considered all measurable forms of fluid intake, such as intravenous and oral fluid administration, minus the fluid output (bleeding, urinary output, and estimated gastrointestinal losses). Insensitive losses were not considered. There was no stablished protocol for fluid restriction, however, the general practice was to restrict fluid. |
| --- |

**Supplementary Table 4.** Patient study power analysis.

| Considering an alpha error of 5% and sample power of 80% with an overall survival rate of 65% for patients with COVID-19 (3) and an 85% survival rate for the group receiving a restrictive fluid balance strategy (4), we estimated a sample size of 154 patients to complete the study (5). |
| --- |

**Table 5**. Comparison of clinical and laboratory data of the collectives at baseline *vs.* D_3_ and between groups at D_3_.

| **Variables** | **CFB**  **negative trend (-)** | **CFB**  **negative trend (-)** |  | | **CFB**  **positive trend (+)** | **CFB**  **positive trend (+)** |  |
| --- | --- | --- | --- | --- | --- | --- | --- |
|  | **D_0_**  **(n=89)** | **D_3_**  **(n=89)** | ***p*** | | **D_0_**  **(n=82)** | **D_3_**  **(n=82)** | ***p*** |
| **SOFA Score**  **mean**±SD | 6.1±2.3 | 5.5±2.2 | **0.027** | | 5.9±2.4 | 6.0±2.5 | 0.0329 |
| median (Q1-Q3) | 6 (4-7) | 5(4-8) |  | | 5 (4-7) | 5(4-7) |  |
| **Creatinine [mg/dL] mean**±SD | 1.12±0.71 | 1.3±0.87 | **0.011** | | 1.4 ±1.4 | 2.1±1.6 | **<0.001** |
| median (Q1-Q3) | 1 (0.75-1.25) | 1.1(0.8-1.6) |  | | 1 (0.8-1.6) | 1.5(0.9-3) |  |
| **Mean arterial pressure**  **mean**±SD | 83.5±15 | 83.0±13.0 | 0.887 | | 81.6±18.1 | 78.0±12.8 | 0.129 |
| median (Q1-Q3) | 81 (74-95) | 85(72-90) |  | | 80(74- 92.7) | 77(71-83) |  |
| **Lactate [mmol/L]**  **mean**±SD | 1.94±0.64 | 2.13±0.56 | **0.007** | | 2.12±0.93 | 2.29±0.7 | 0.094 |
| median (Q1-Q3) | 1.74 (1.52-2.3) | 2.08(1.8-2.5) |  | | 1.90 (1.56-2.43) | 2.3(1.8-2.6) |  |
| **Hemoglobin [g/dL]**  **mean**±SD | 12.3±1.87 | 11.78±1.56 | **0.008** | | 12.2±2.0 | 11.19±2.0 | **0.006** |
| median (Q1-Q3) | 12.5 (11.4-13.5) | 12(10.7-13.0) |  | | 12 (11-13.7) | 11.6 (9.4-12.7) |  |
| **Comparison of D_3_ *vs*. D_3_** | | | | | | | |
| **SOFA Score**  **mean**±SD | 5.5±2.2 | | |  | 6.0±2.5 | | 0.208 |
| median (Q1-Q3) | 5(4-8) | | |  | 5(4-7) | |  |
| **Creatinine [mg/dL]**  **mean**±SD | 1.3±0.87 | | |  | 2.1±1.6 | | **0.002** |
| median (Q1-Q3) | 1.1(0.8-1.6) | | |  | 1.5(0.9-3) | |  |
| **Mean arterial pressure**  **mean**±SD | 83.0±13.0 | | |  | 78.0±12.8 | | **0.008** |
| median (Q1-Q3) | 85(72-90) | | |  | 77(71-83) | |  |
| **Lactate [mmol/L]**  **mean**±SD | 2.13±0.56 | | |  | 2.29±0.7 | | 0.386 |
| median (Q1-Q3) | 2.08(1.8-2.5) | | |  | 2.3(1.8-2.6) | |  |
| **Hemoglobin [g/dL]**  **mean**±SD | 11.78±1.56 | | |  | 11.19±2.0 | | 0.187 |
| median (Q1-Q3) | 12(10.7-13.0) | | |  | 11.6 (9.4-12.7) | |  |
| **Cumulative Fluid Balance trend [ml]**  **mean**±SD | 181.4± 686.2 | | |  | 718±817.5 | | **<0.001** |
| median (Q1-Q3) | 238(-294-628) | | |  | 572(90-1303) | |  |

*Values summarized as* ***mean±SD and median*** *Q1/3 (first and third quartile).p-value was calculated by Wilcoxon signed rank test for the comparison between baseline and D_3_ continuous variables of each group. The comparison von D_3_ vs. D_3_ was mad with Mann-Whitney test. SOFA Score: Sequential Organ Failure Assessment Score; PaO_2_: partial pressure of oxygen in the arterial blood; PEEP: Positive end-expiratory pressure; p: p-value (<0.05 marked in bold).*

**Supplementary Figure 1.** Cumulative fluid balance trend based on the divided groups after the model-based clustering visual distribution of clusters using dispersion.


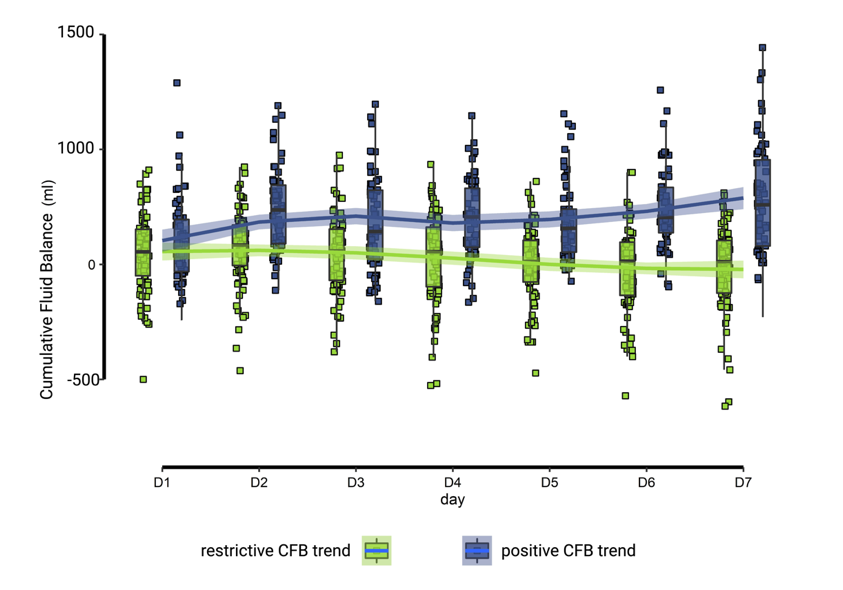


Shows the allocation of groups using model-based clustering. The graph shows the CFB trend of each group from their baselines to D_7_. CFB: Cumulative Fluid Balance, Q1/3 (first and third quartile).

**Supplementary Figure 2.** Example of vector trajectory for clustering using k-mean Trend.


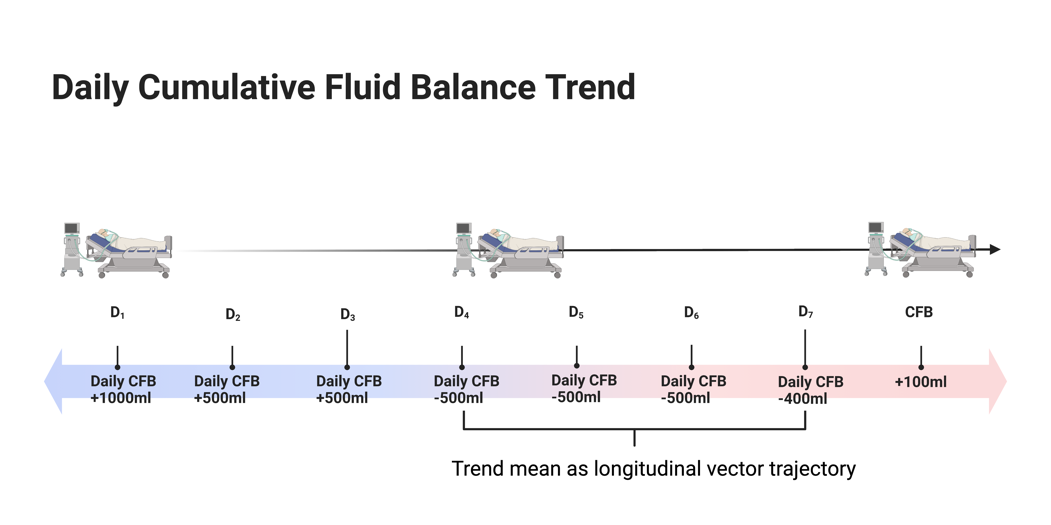


CFB= cumulative fluid balance.

**Supplementary Figure 3.** Cumulative fluid balance trend based on the divided groups after the model-based clustering.


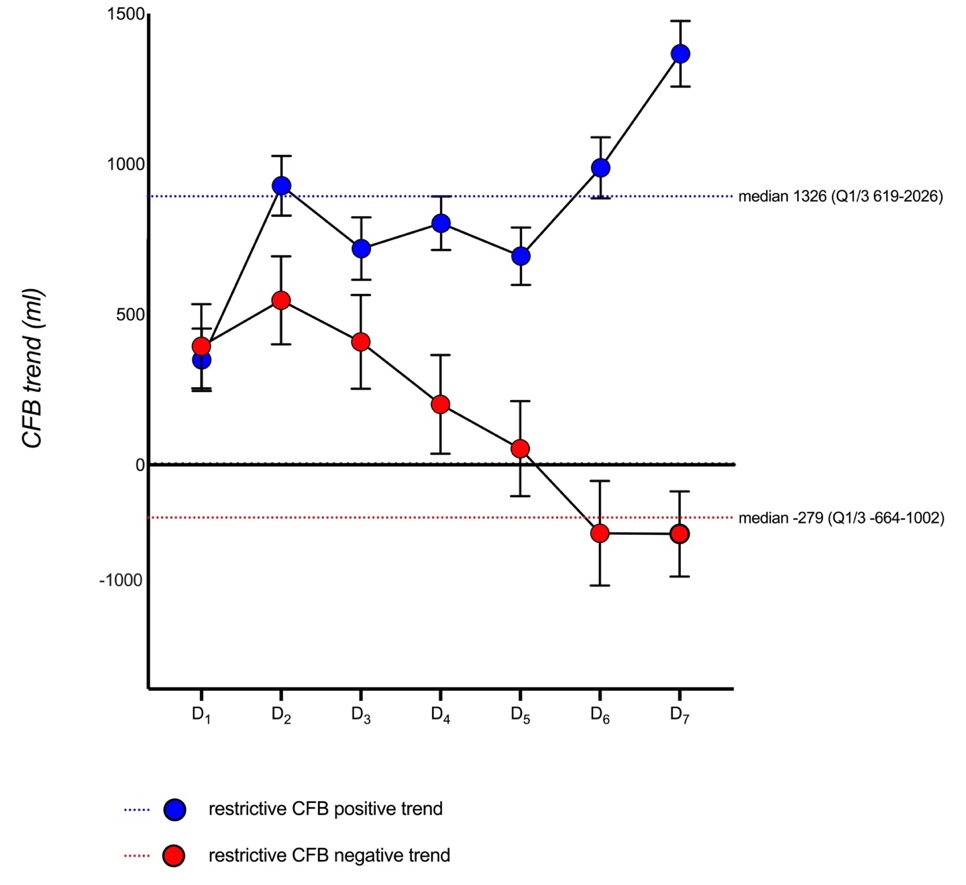


Shows the allocation of groups using model-based clustering. The graph shows the CFB trend of each group from their baselines to D_7_. CFB: Cumulative Fluid Balance, Q1/3 (first and third quartile).

**Supplementary Figure 4.** Kaplan-Meier with the estimated cumulative probability of 28-day survival using CFB trend at D_3_ as independent variable.


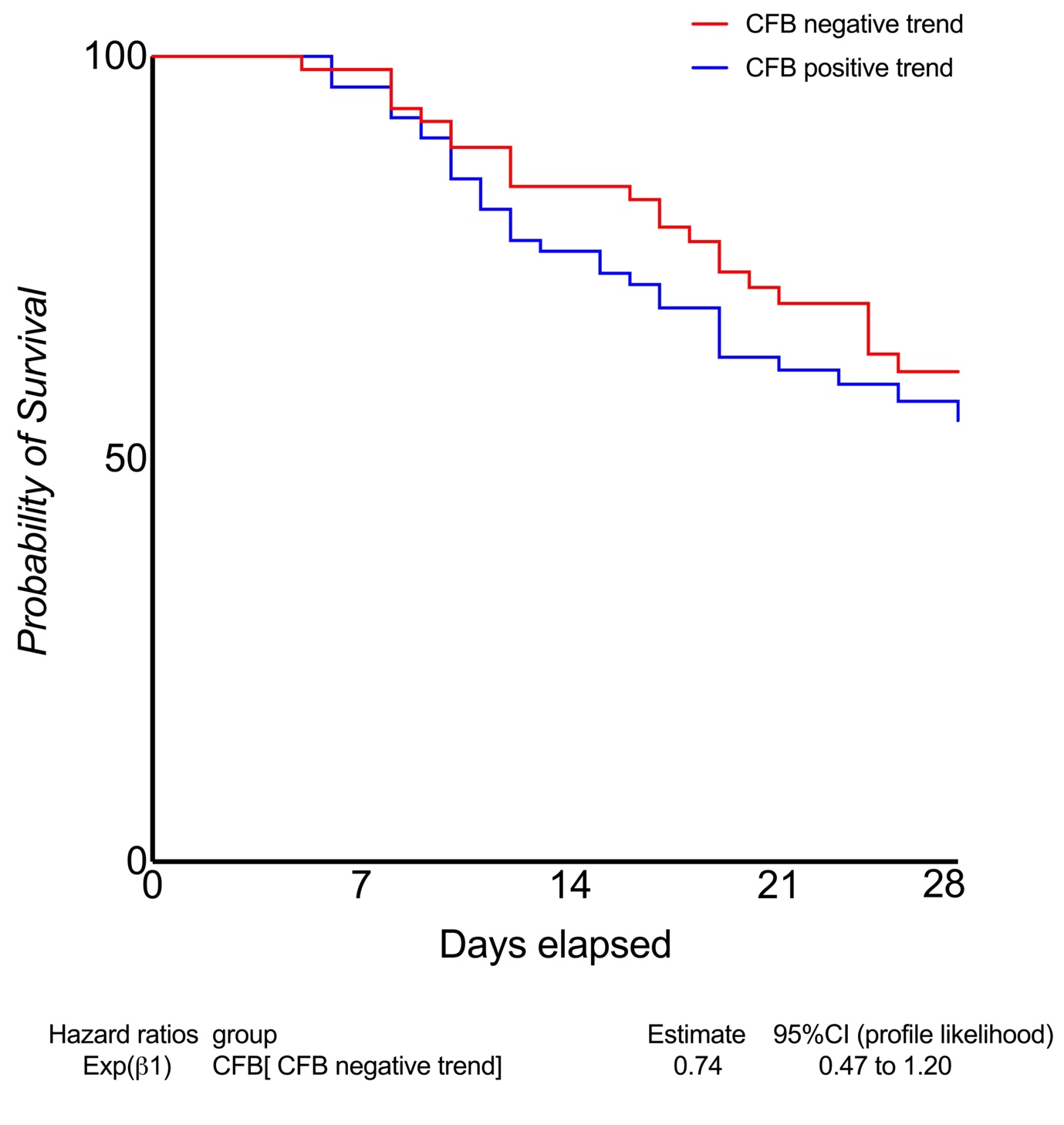


**Supplementary References**

1. Ferguson ND, Fan E, Camporota L, et al: The Berlin definition of ARDS: an expanded rationale, justification, and supplementary material. *Intensive Care Medicine* 2012; 38(10):1573-1582

2. Khwaja A: KDIGO clinical practice guidelines for acute kidney injury. *Nephron Clin Pract* 2012; 120(4):c179-184

3. Perazzo H, Cardoso SW, Ribeiro MPD, et al: In-hospital mortality and severe outcomes after hospital discharge due to COVID-19: A prospective multicenter study from Brazil. *The Lancet Regional Health – Americas* 2022; 11

4. Ahuja S, de Grooth H-J, Paulus F, et al: Association between early cumulative fluid balance and successful liberation from invasive ventilation in COVID-19 ARDS patients — insights from the PRoVENT-COVID study: a national, multicenter, observational cohort analysis. *Critical Care* 2022; 26(1):157

5. Walters SJ, Jacques RM, Dos Anjos Henriques-Cadby IB, et al: Sample size estimation for randomised controlled trials with repeated assessment of patient-reported outcomes: what correlation between baseline and follow-up outcomes should we assume? *Trials* 2019; 20(1):566
